# First person – Yujiao Wang

**DOI:** 10.1242/dmm.052192

**Published:** 2024-11-27

## Abstract

First Person is a series of interviews with the first authors of a selection of papers published in Disease Models & Mechanisms, helping researchers promote themselves alongside their papers. Yujiao Wang is first author on ‘ A high-fat plus high-sucrose diet induces age-related macular degeneration in an experimental rabbit model’, published in DMM. Yujiao is a Lecturer and Ophthalmologist in the lab of Danian Chen at West China Hospital, Sichuan University, investigating Animal models of ocular disease (retinal disease and orbital disease).


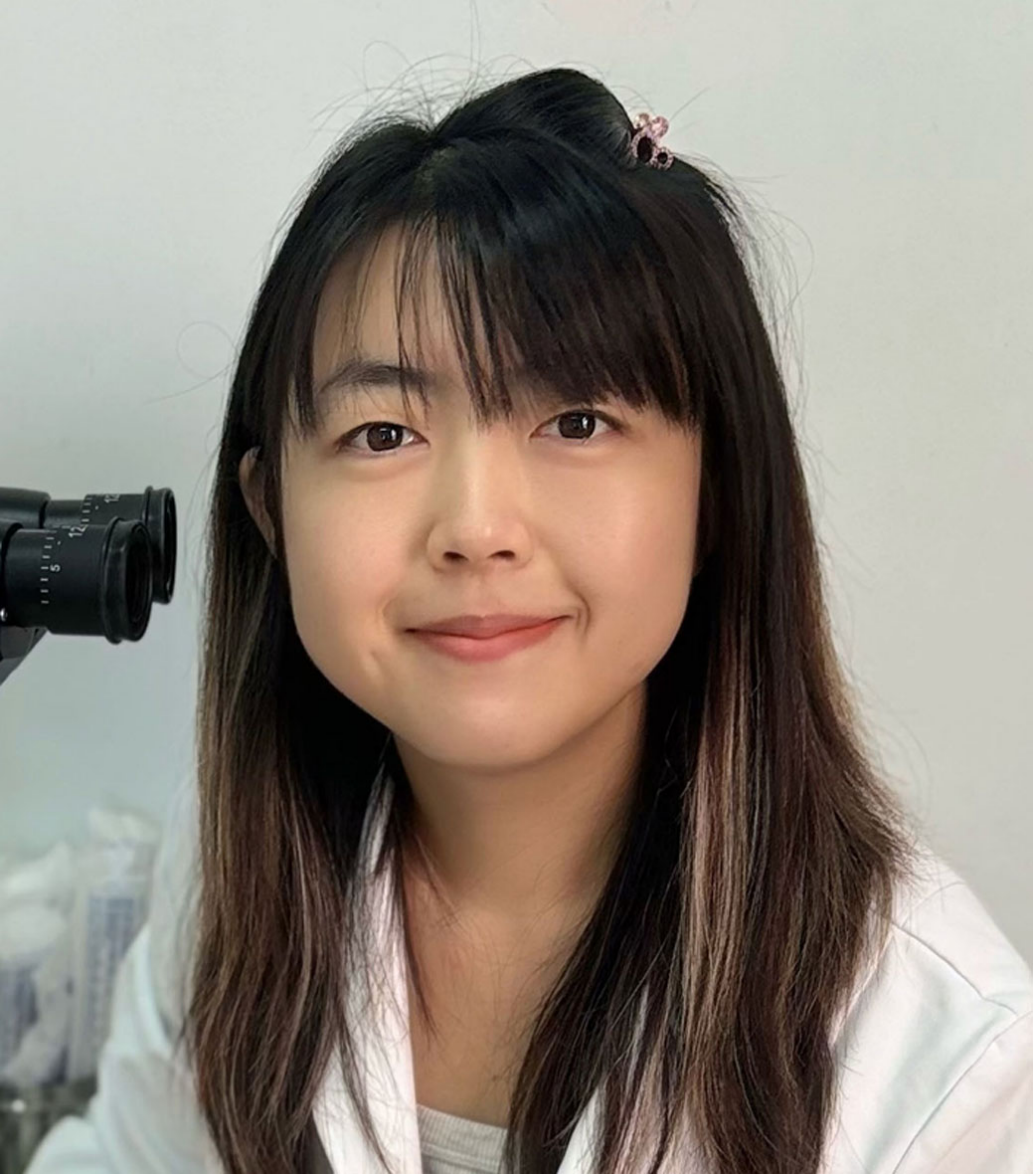


**Yujiao Wang**

**Who or what inspired you to become a scientist?**

Since childhood, I have been curious about the human body and medicine. My mother was a radiologist. In my spare time, she always took me to her place of work, and I always tirelessly asked her questions about human organs in ultrasonic images. Instead of telling me the answers, she encouraged me to find them myself. After high school, I went to Medical College and interned at a hospital, witnessing patients being diagnosed with diseases that had no cure. These experiences inspired me to become a disease scientist. Later, my PhD supervisor, Danian Chen, with his stringent careful attitude towards scientific work, further motivated and encouraged me to be a disease scientist.

Diet is one of the most important environmental risk factors for AMD

**What is the main question or challenge in disease biology you are addressing in this paper? How did you go about investigating your question or challenge?**

Age-related macular degeneration (AMD), including its dry and wet forms, is a leading cause of vision loss in older people. There is no treatment for dry AMD which affects most AMD patients. Before our study, rabbit has never been used as a model for dry AMD. Diet is one of the most important environmental risk factors for AMD. High intake of cholesterol and saturated fat have long been considered critical in the development of AMD, although, some recent studies reported no significant associations between serum lipoprotein profiles and AMD (Lüdtke et al., 2019; Mao et al., 2019). Whether systemic lipid profiles directly influence the lipid metabolism of retina and AMD development is unknown, which is why we tested whether a combined a high-fat high-sugar diet would induce AMD in rabbits. We found this strategy successful in inducing dry AMD in rabbits.

**How would you explain the main findings of your paper to non-scientific family and friends?**

The eye is a delicate organ that functions like a camera. The retina, its main structure at the back of the eyeball, functions as the film and the macular area, the central part of the retina, has the best visual ability. AMD is a retinal disease that damages the retinal macular, leading to vision loss. AMD has two stages: early or dry AMD and late or wet AMD. There is no cure for dry AMD right now. Animal models are fundamental for studying human eye diseases and discovering new therapeutic targets. Rodents are commonly used animal models, and many studies used rodents that have been fed a high-glycemic index (GI) diet to induce many AMD features. As herbivorous animals, rabbits, are susceptible to high-fat diets and a useful model to study hyperlipidemia. In addition, the eye anatomy of rabbits is similar to that of humans. Thus, we compared lipid profiles and retinal phenotypes between groups of male Chinchilla rabbits that had long-term been fed four different diets. We found that feeding a high-fat plus high-sucrose diet (HFSD) induces dry AMD-like retinal lesions.

**What are the potential implications of these results for disease biology and the possible impact on patients?**

AMD is a leading cause of blindness, which is associated with metabolic disorders and diets. Our study used chinchilla rabbits to mimic hyperlipidemia disorders in humans. The results showed that feeding a high-fat diet (HFD) or high-sucrose diet (HSD) had only minor effects on lipid profiles but that a HFSD was able to induce severe dyslipidemia. Moreover, a HFSD can induce retinal lesions, such as reticular pseudo-drusen (RPDs), and pigmentary abnormalities in rabbits (Wang et al., 2024). So, HFSD-fed male Chinchilla rabbits are a good model of early AMD, and use of this animal model might significantly assist in finding early pathological features of AMD, revealing the biochemical and genetic perturbations that occur during the disease and developing new therapies to treat patients diagnosed with AMD at an earlier stage.

**Why did you choose DMM for your paper?**

DMM is a highly regarded research journal with a strong focus on animal models. We are excited that our work is published in this journal.

**Given your current role, what challenges do you face and what changes could improve the professional lives of other scientists in this role?**

I am a young female clinician and scientist. It seems that the biggest challenges for us are funding, time, research teams and family. The competition for national-level funding is very strong in China, and the funding rate is about only 10%. My life is always busy. I also try to establish a stable research team and to secure sufficient lab space. I hope there will be some increase in funding, so more students can come and work with me.

**What's next for you?**

As a lecturer and ophthalmologist at a large hospital, I am particularly interested in using rabbits and rodents as disease models to study the pro-inflammatory role of retinal microglia, and the interaction between microglia and Müller cells in the retina. I am currently using multi-omics analyses − including single-cell sequencing and spatial transcriptome sequencing technologies − to study these topics using this rabbit model. I am also collaborating with experts in nanotechnology to find drug-delivery strategies in order to treat AMD and other ocular diseases.

**Tell us something interesting about yourself that wouldn't be on your CV**

I love hiking, climbing mountains and go for a run daily. I prefer to choose my favorite cities to run half marathons. The scenery along the route and climbing to every top excites and comforts me.
